# Complete genome sequence of *Prevotella bivia* GTC18476: a clinical isolate with reduced susceptibility to metronidazole in Japan

**DOI:** 10.1128/mra.00688-26

**Published:** 2026-08-21

**Authors:** Masahiro Hayashi, Tatsuya Natori, Yoshinori Muto, Ayaka Kasai, Tomu Kamijo, Taku Yamane, Ayaka Hachiro, Kaori Tanaka, Nau Ishimine, Takeshi Uehara

**Affiliations:** 1 Institute for Glyco-core Research iGCORE, Gifu University 12785, Gifu City, Gifu, Japan; 2 Division of Anaerobe Research, Life Science Research Center, Gifu University 12785, Gifu City, Gifu, Japan; 3 Gifu University Center for Conservation of Microbial Genetic Resource 12785, Gifu City, Gifu, Japan; 4 United Graduate School of Drug Discovery and Medical Information Sciences, Gifu University 12785, Gifu City, Gifu, Japan; 5 Department of Laboratory Medicine, Shinshu University Hospital 118108, Matsumoto, Japan; University of Pittsburgh School of Medicine, Pittsburgh, Pennsylvania, USA

**Keywords:** *Prevotella bivia*, anaerobes, *nimK*, genomes

## Abstract

Here, we report the complete genome sequence of *Prevotella bivia* GTC 18476, isolated from a clinical specimen in Nagano, Japan. The genome comprises two circular chromosomes and one plasmid measuring 1,298,955 bp, 1,212,313 bp, and 19,020 bp, respectively.

## ANNOUNCEMENT

*Prevotella bivia* is an anaerobic gram-negative rod associated with opportunistic infections, and antimicrobial resistance among anaerobes is increasingly prevalent (1, 2). Strain GTC 18476 was isolated from a purulent umbilical scar infection in an adult patient in Nagano, Japan. The isolate was cultured on Anaero Columbia RS Blood Agar (Nippon Becton Dickinson, Japan) at 37°C for 48 h under anaerobic conditions (82% N_2_, 10% CO_2_, and 8% H_2_). Species identification was confirmed by matrix-assisted laser desorption/ionization time-of-flight mass spectrometry (Bruker, Germany). Antimicrobial susceptibility testing of GTC18476 (dry plate method; Eiken DP53, Tokyo, Japan) showed a metronidazole MIC of >16 µg/mL.

For genomic extraction, the strain was cultured under the same conditions. Cells were harvested, suspended in Tris-EDTA buffer, and pelleted by centrifugation, and genomic DNA was extracted using the NucleoBond HMW DNA Kit (Macherey-Nagel, Japan).

The genome was sequenced using PromethION 2i (Oxford Nanopore Technologies [ONT], Oxford, UK) for long-read sequencing and DNBSEQ (MGITech Co., Ltd., Shenzhen, China) for short-read sequencing (3–5). For long-read sequencing, a library was prepared using the Native Barcoding Kit (SQK-LSK114-24; ONT) without DNA shearing. Short DNA fragments were removed using the Short Read Eliminator XS Kit (Pacific Biosciences of California, Inc., USA). Sequencing was performed using ONT with a FLO-PRO114M flow cell. Base calling was performed using Dorado v.7.2.14 (super-accurate model), and reads were trimmed and quality-filtered using NanoFilt v.2.7.1 (6) with the parameters “-l 1000 -q 10 --headcrop 50.” For short-read sequencing, libraries were prepared using the MGIEasy FS DNA Library Prep Set (MGI Tech), followed by 2 × 150 bp paired-end sequencing on the DNBSEQ-G400RS platform (MGI Tech). Reads were processed with fastp v0.20.1 (7) with the parameters “-q 30 -n 20 -t 1 -T 1.” Short-read quality was evaluated using fastp, and long-read quality was assessed using NanoPlot v1.32.1 (7). Short reads (>95.0% of bases >Q30) and long reads (mean read quality of 12.7) were assembled using Unicycler v.0.4.8 (8) with default settings. Assembly circularization was performed automatically using Unicycler. The assembly graph was visualized using Bandage v0.8.1 (9), and taxonomic integrity was confirmed using Blobtools v1.0 with short-read data (10). Genome annotation was performed using the DDBJ Fast Annotation and Submission Tool (DFAST) (11). The completed assembly was rotated to begin with the *dnaA* gene on the forward strand.

Genomic characteristics are summarized in Table 1. Unicycler generated a single circular genome, and circularization was confirmed by Bandage. Genome quality and assembly statistics were assessed using CheckM v1.2.2 (12) and SeqKit v2.9.0 (13). CheckM estimated 100.0% completeness and 0.0% contamination. DFAST predicted 2,088 coding sequences, including 12 rRNAs, 50 tRNAs, and 0 CRISPR loci. It also identified a *nimK* gene on chromosome 1, which may contribute to the reduced susceptibility to metronidazole.

**TABLE 1:** Complete genome characteristics of *Prevotella bivia* GTC 18476 isolated from a clinical specimen in Japan

Parameter	Strain name
	*Prevotella bivia* GTC 18476
DNBSEQ sequencing^*a*^	
No. of reads	4,368,978
Size (kb)	655,346.7
Avg coverage (×)	259.0
DRA accession no.	DRR628306
ONT sequencing^*a*^	
No. of reads	73,153
Size (kb)	860,426
Avg read length (bp)	11,762
Avg coverage (×)	340.5
N50	17,227.0
DRA accession no.	DRR628307
Assembly	
Assembly N50 (bp)^*b*^	1,298,955
Estimated genome completeness (%)^*c*^	100.0
Estimated genome contamination (%)^*c*^	0.0
Genome structure	Two chromosomes, one plasmid
DDBJ/GenBank accession no.	Chromosome 1 (AP038911)
	Chromosome 2 (AP038912)
	Plasmid (AP038913)
Genome size (bp)	1,298,955 (chromosome 1)
	1,212,313 (chromosome 2)
	19,020 (Plasmid)
GC content (%) (chromosome/plasmid name)	39.4 (chromosome 1)
	39.4 (chromosome 2)
	33.2 (Plasmid)
No. of coding sequences^*d*^	2,088
No. of rRNAs^*d*^	12
No. of tRNAs^*d*^	50
No. of CRISPRs^*d*^	0

^*a*^ DRA, DDBJ Sequence Read Archive.

^*b*^ Determined with seqkit v2.9.0.

^*c*^ Determined with CheckM v1.2.2.

^*d*^ DFAST, DDBJ Fast Annotation and Submission Tool.

## Data Availability

The genome sequence of *Prevotella bivia* GTC18476 has been deposited in DDBJ (https://www.ddbj.nig.ac.jp/index.html) under accession numbers AP038911, AP038912, and AP038913 for chromosome 1, chromosome 2, and plasmid pGTC18476, respectively. Raw sequencing data for GTC18476 have been deposited in the DDBJ Sequence Read Archive under accession numbers DRR628306 and DRR628307.
